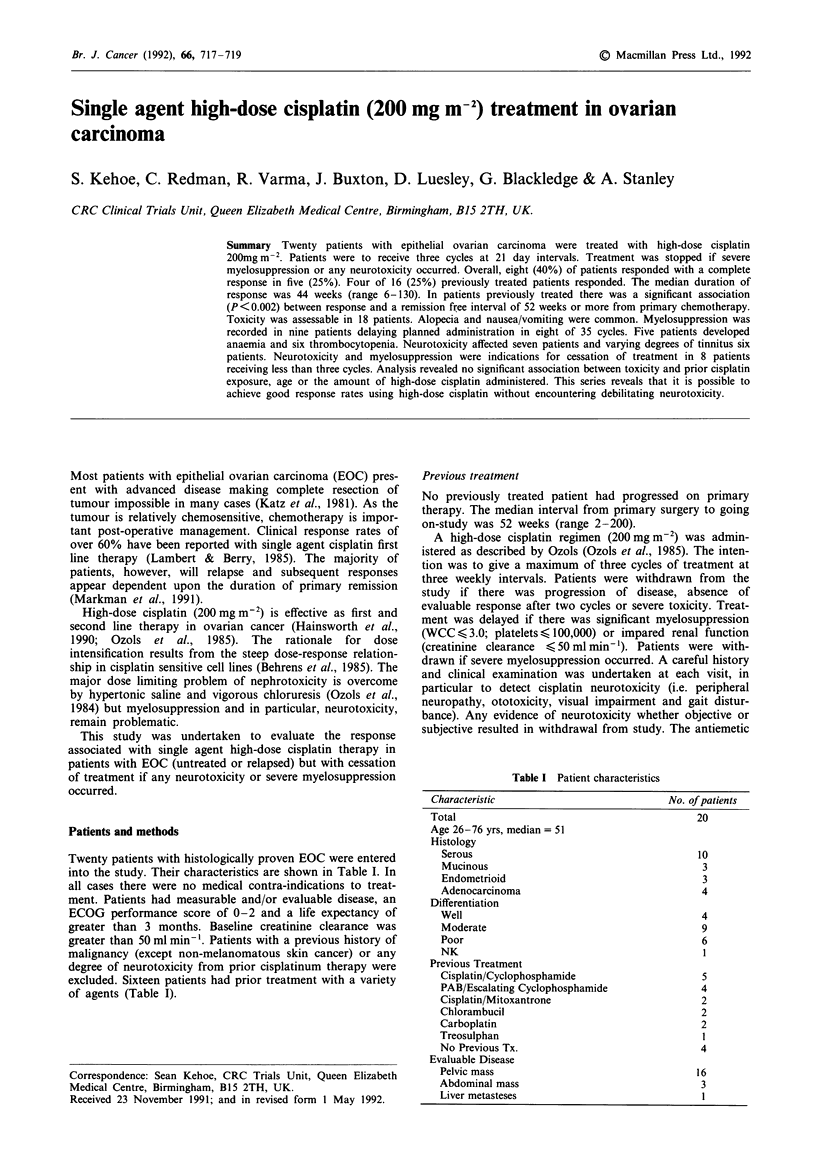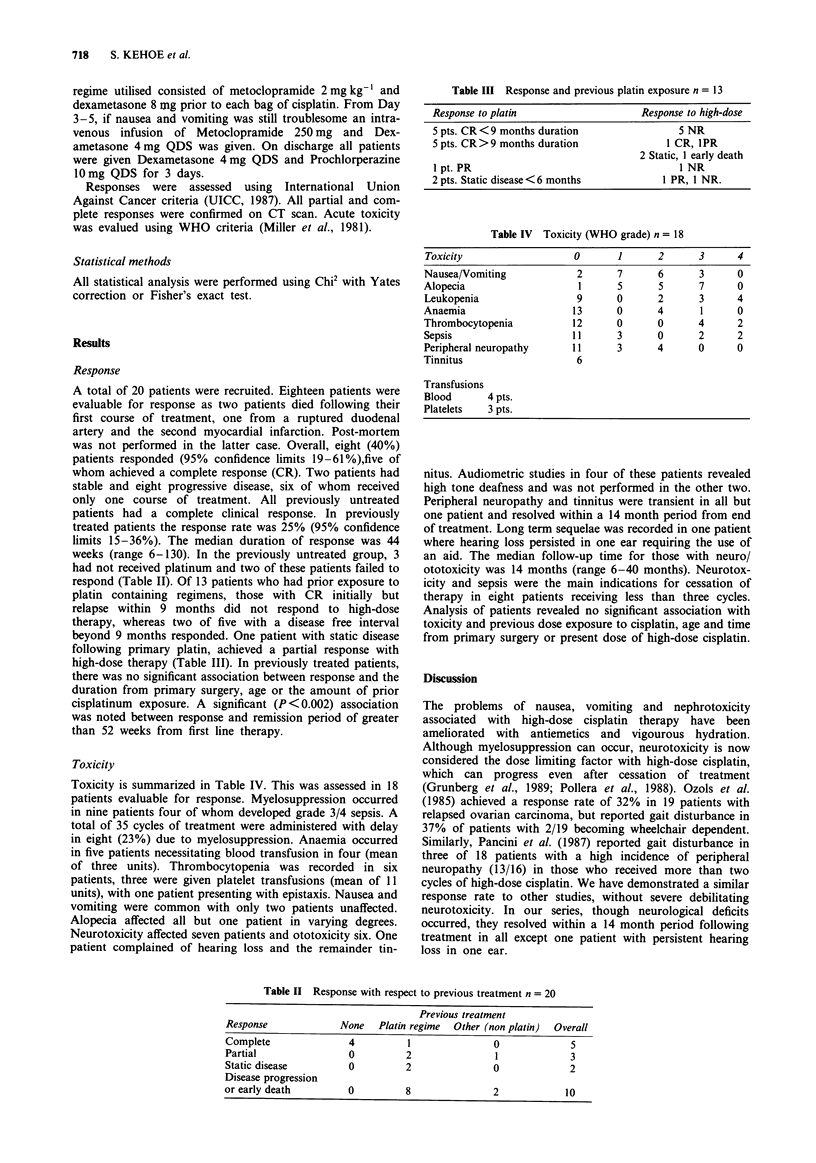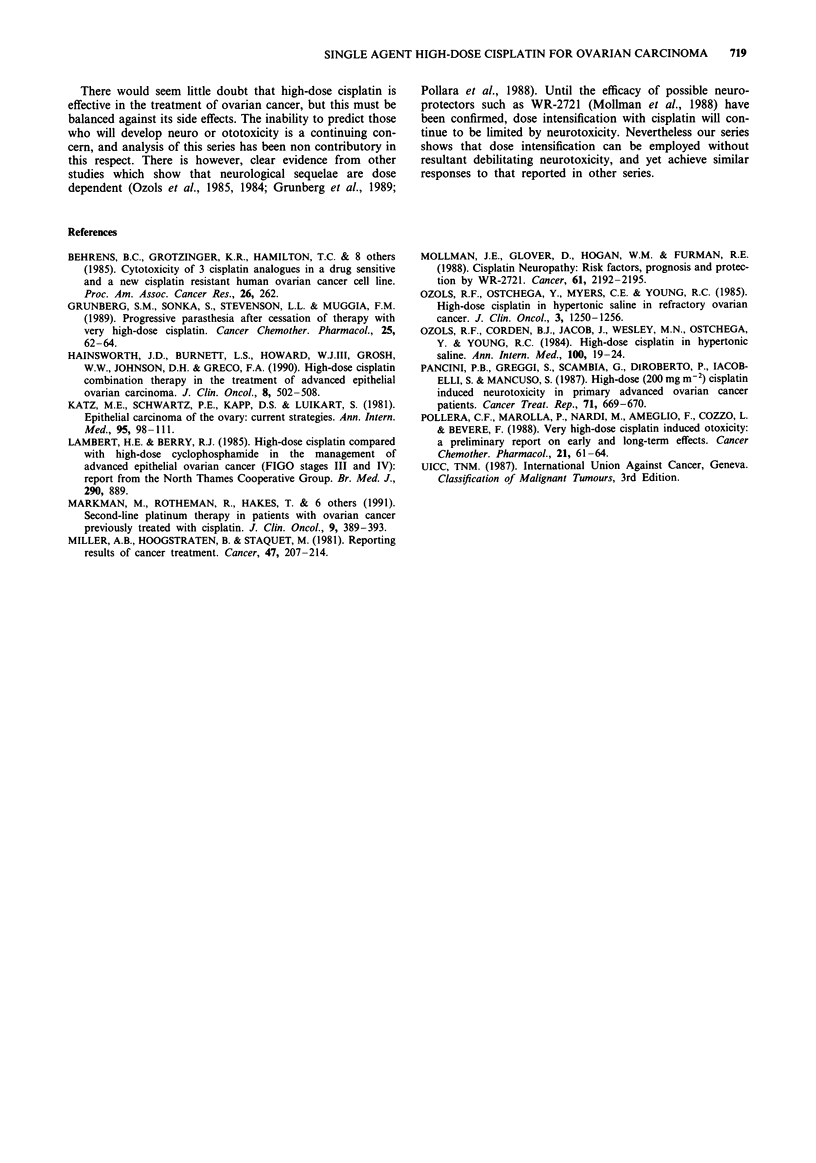# Single agent high-dose cisplatin (200 mg m-2) treatment in ovarian carcinoma.

**DOI:** 10.1038/bjc.1992.344

**Published:** 1992-10

**Authors:** S. Kehoe, C. Redman, R. Varma, J. Buxton, D. Luesley, G. Blackledge, A. Stanley

**Affiliations:** CRC Clinical Trials Unit, Queen Elizabeth Medical Centre, Birmingham, UK.

## Abstract

Twenty patients with epithelial ovarian carcinoma were treated with high-dose cisplatin 200mg m-2. Patients were to receive three cycles at 21 day intervals. Treatment was stopped if severe myelosuppression or any neurotoxicity occurred. Overall, eight (40%) of patients responded with a complete response in five (25%). Four of 16 (25%) previously treated patients responded. The median duration of response was 44 weeks (range 6-130). In patients previously treated there was a significant association (P < 0.002) between response and a remission free interval of 52 weeks or more from primary chemotherapy. Toxicity was assessable in 18 patients. Alopecia and nausea/vomiting were common. Myelosuppression was recorded in nine patients delaying planned administration in eight of 35 cycles. Five patients developed anaemia and six thrombocytopenia. Neurotoxicity affected seven patients and varying degrees of tinnitus six patients. Neurotoxicity and myelosuppression were indications for cessation of treatment in 8 patients receiving less than three cycles. Analysis revealed no significant association between toxicity and prior cisplatin exposure, age or the amount of high-dose cisplatin administered. This series reveals that it is possible to achieve good response rates using high-dose cisplatin without encountering debilitating neurotoxicity.


Br. J. Cancer (1992), 66, 717-719 © Macmillan Press Ltd., 1992

### Single agent high-dose cisplatin (200 mg $m^{-2}$) treatment in ovarian carcinoma

S. Kehoe, C. Redman, R. Varma, J. Buxton, D. Luesley, G. Blackledge & A. Stanley

*CRC Clinical Trials Unit, Queen Elizabeth Medical Centre, Birmingham, B15 2TH, UK.*

**Summary** Twenty patients with epithelial ovarian carcinoma were treated with high-dose cisplatin 200mg $m^{-2}$. Patients were to receive three cycles at 21 day intervals. Treatment was stopped if severe myelosuppression or any neurotoxicity occurred. Overall, eight (40%) of patients responded with a complete response in five (25%). Four of 16 (25%) previously treated patients responded. The median duration of response was 44 weeks (range 6-130). In patients previously treated there was a significant association ($P < 0.002$) between response and a remission free interval of 52 weeks or more from primary chemotherapy. Toxicity was assessable in 18 patients. Alopecia and nausea/vomiting were common. Myelosuppression was recorded in nine patients delaying planned administration in eight of 35 cycles. Five patients developed anaemia and six thrombocytopenia. Neurotoxicity affected seven patients and varying degrees of tinnitus six patients. Neurotoxicity and myelosuppression were indications for cessation of treatment in 8 patients receiving less than three cycles. Analysis revealed no significant association between toxicity and prior cisplatin exposure, age or the amount of high-dose cisplatin administered. This series reveals that it is possible to achieve good response rates using high-dose cisplatin without encountering debilitating neurotoxicity.

Most patients with epithelial ovarian carcinoma (EOC) present with advanced disease making complete resection of tumour impossible in many cases (Katz *et al.*, 1981). As the tumour is relatively chemosensitive, chemotherapy is important post-operative management. Clinical response rates of over 60% have been reported with single agent cisplatin first line therapy (Lambert & Berry, 1985). The majority of patients, however, will relapse and subsequent responses appear dependent upon the duration of primary remission (Markman *et al.*, 1991).

High-dose cisplatin (200 mg $m^{-2}$) is effective as first and second line therapy in ovarian cancer (Hainsworth *et al.*, 1990; Ozols *et al.*, 1985). The rationale for dose intensification results from the steep dose-response relationship in cisplatin sensitive cell lines (Behrens *et al.*, 1985). The major dose limiting problem of nephrotoxicity is overcome by hypertonic saline and vigorous chloruresis (Ozols *et al.*, 1984) but myelosuppression and in particular, neurotoxicity, remain problematic.

This study was undertaken to evaluate the response associated with single agent high-dose cisplatin therapy in patients with EOC (untreated or relapsed) but with cessation of treatment if any neurotoxicity or severe myelosuppression occurred.

### Patients and methods

Twenty patients with histologically proven EOC were entered into the study. Their characteristics are shown in Table I. In all cases there were no medical contra-indications to treatment. Patients had measurable and/or evaluable disease, an ECOG performance score of 0-2 and a life expectancy of greater than 3 months. Baseline creatinine clearance was greater than 50 ml $min^{-1}$. Patients with a previous history of malignancy (except non-melanomatous skin cancer) or any degree of neurotoxicity from prior cisplatinum therapy were excluded. Sixteen patients had prior treatment with a variety of agents (Table I).

*Previous treatment*

No previously treated patient had progressed on primary therapy. The median interval from primary surgery to going on-study was 52 weeks (range 2-200).

A high-dose cisplatin regimen (200 mg $m^{-2}$) was administered as described by Ozols (Ozols *et al.*, 1985). The intention was to give a maximum of three cycles of treatment at three weekly intervals. Patients were withdrawn from the study if there was progression of disease, absence of evaluable response after two cycles or severe toxicity. Treatment was delayed if there was significant myelosuppression (WCC $\leqslant 3.0$; platelets $\leqslant 100,000$) or impared renal function (creatinine clearance $\leqslant 50$ ml $min^{-1}$). Patients were withdrawn if severe myelosuppression occurred. A careful history and clinical examination was undertaken at each visit, in particular to detect cisplatin neurotoxicity (i.e. peripheral neuropathy, ototoxicity, visual impairment and gait disturbance). Any evidence of neurotoxicity whether objective or subjective resulted in withdrawal from study. The antiemetic

**Table I** Patient characteristics

| *Characteristic* | *No. of patients* |
|---|---|
| Total | 20 |
| Age 26-76 yrs, median = 51 | |
| Histology | |
| Serous | 10 |
| Mucinous | 3 |
| Endometrioid | 3 |
| Adenocarcinoma | 4 |
| Differentiation | |
| Well | 4 |
| Moderate | 9 |
| Poor | 6 |
| NK | 1 |
| Previous Treatment | |
| Cisplatin/Cyclophosphamide | 5 |
| PAB/Escalating Cyclophosphamide | 4 |
| Cisplatin/Mitoxantrone | 2 |
| Chlorambucil | 2 |
| Carboplatin | 2 |
| Treosulphan | 1 |
| No Previous Tx. | 4 |
| Evaluable Disease | |
| Pelvic mass | 16 |
| Abdominal mass | 3 |
| Liver metasteses | 1 |

Correspondence: Sean Kehoe, CRC Trials Unit, Queen Elizabeth Medical Centre, Birmingham, B15 2TH, UK.
Received 23 November 1991; and in revised form 1 May 1992.

regime utilised consisted of metoclopramide 2 mg kg$^{-1}$ and dexametasone 8 mg prior to each bag of cisplatin. From Day 3–5, if nausea and vomiting was still troublesome an intravenous infusion of Metoclopramide 250 mg and Dexametasone 4 mg QDS was given. On discharge all patients were given Dexametasone 4 mg QDS and Prochlorperazine 10 mg QDS for 3 days.

Responses were assessed using International Union Against Cancer criteria (UICC, 1987). All partial and complete responses were confirmed on CT scan. Acute toxicity was evalued using WHO criteria (Miller *et al*., 1981).

### *Statistical methods*

All statistical analysis were performed using Chi$^2$ with Yates correction or Fisher's exact test.

### Results

### *Response*

A total of 20 patients were recruited. Eighteen patients were evaluable for response as two patients died following their first course of treatment, one from a ruptured duodenal artery and the second myocardial infarction. Post-mortem was not performed in the latter case. Overall, eight (40%) patients responded (95% confidence limits 19–61%),five of whom achieved a complete response (CR). Two patients had stable and eight progressive disease, six of whom received only one course of treatment. All previously untreated patients had a complete clinical response. In previously treated patients the response rate was 25% (95% confidence limits 15–36%). The median duration of response was 44 weeks (range 6–130). In the previously untreated group, 3 had not received platinum and two of these patients failed to respond (Table II). Of 13 patients who had prior exposure to platin containing regimens, those with CR initially but relapse within 9 months did not respond to high-dose therapy, whereas two of five with a disease free interval beyond 9 months responded. One patient with static disease following primary platin, achieved a partial response with high-dose therapy (Table III). In previously treated patients, there was no significant association between response and the duration from primary surgery, age or the amount of prior cisplatinum exposure. A significant ($P < 0.002$) association was noted between response and remission period of greater than 52 weeks from first line therapy.

### *Toxicity*

Toxicity is summarized in Table IV. This was assessed in 18 patients evaluable for response. Myelosuppression occurred in nine patients four of whom developed grade 3/4 sepsis. A total of 35 cycles of treatment were administered with delay in eight (23%) due to myelosuppression. Anaemia occurred in five patients necessitating blood transfusion in four (mean of three units). Thrombocytopenia was recorded in six patients, three were given platelet transfusions (mean of 11 units), with one patient presenting with epistaxis. Nausea and vomiting were common with only two patients unaffected. Alopecia affected all but one patient in varying degrees. Neurotoxicity affected seven patients and ototoxicity six. One patient complained of hearing loss and the remainder tinnitus. Audiometric studies in four of these patients revealed high tone deafness and was not performed in the other two. Peripheral neuropathy and tinnitus were transient in all but one patient and resolved within a 14 month period from end of treatment. Long term sequelae was recorded in one patient where hearing loss persisted in one ear requiring the use of an aid. The median follow-up time for those with neuro/ototoxicity was 14 months (range 6–40 months). Neurotoxicity and sepsis were the main indications for cessation of therapy in eight patients receiving less than three cycles. Analysis of patients revealed no significant association with toxicity and previous dose exposure to cisplatin, age and time from primary surgery or present dose of high-dose cisplatin.

**Table III** Response and previous platin exposure $n = 13$

| *Response to platin* | *Response to high-dose* |
|---|---|
| 5 pts. CR < 9 months duration | 5 NR |
| 5 pts. CR > 9 months duration | 1 CR, 1PR<br>2 Static, 1 early death |
| 1 pt. PR | 1 NR |
| 2 pts. Static disease < 6 months | 1 PR, 1 NR. |

**Table IV** Toxicity (WHO grade) $n = 18$

| *Toxicity* | *0* | *1* | *2* | *3* | *4* |
|---|---|---|---|---|---|
| Nausea/Vomiting | 2 | 7 | 6 | 3 | 0 |
| Alopecia | 1 | 5 | 5 | 7 | 0 |
| Leukopenia | 9 | 0 | 2 | 3 | 4 |
| Anaemia | 13 | 0 | 4 | 1 | 0 |
| Thrombocytopenia | 12 | 0 | 0 | 4 | 2 |
| Sepsis | 11 | 3 | 0 | 2 | 2 |
| Peripheral neuropathy | 11 | 3 | 4 | 0 | 0 |
| Tinnitus | 6 | | | | |
| Transfusions | | | | | |
| Blood 4 pts. | | | | | |
| Platelets 3 pts. | | | | | |

### Discussion

The problems of nausea, vomiting and nephrotoxicity associated with high-dose cisplatin therapy have been ameliorated with antiemetics and vigourous hydration. Although myelosuppression can occur, neurotoxicity is now considered the dose limiting factor with high-dose cisplatin, which can progress even after cessation of treatment (Grunberg *et al*., 1989; Pollera *et al*., 1988). Ozols *et al.* (1985) achieved a response rate of 32% in 19 patients with relapsed ovarian carcinoma, but reported gait disturbance in 37% of patients with 2/19 becoming wheelchair dependent. Similarly, Pancini *et al.* (1987) reported gait disturbance in three of 18 patients with a high incidence of peripheral neuropathy (13/16) in those who received more than two cycles of high-dose cisplatin. We have demonstrated a similar response rate to other studies, without severe debilitating neurotoxicity. In our series, though neurological deficits occurred, they resolved within a 14 month period following treatment in all except one patient with persistent hearing loss in one ear.

**Table II** Response with respect to previous treatment $n = 20$

| *Response* | *Previous treatment* *None* | *Platin regime* | *Other (non platin)* | *Overall* |
|---|---|---|---|---|
| Complete | 4 | 1 | 0 | 5 |
| Partial | 0 | 2 | 1 | 3 |
| Static disease | 0 | 2 | 0 | 2 |
| Disease progression or early death | 0 | 8 | 2 | 10 |

There would seem little doubt that high-dose cisplatin is effective in the treatment of ovarian cancer, but this must be balanced against its side effects. The inability to predict those who will develop neuro or ototoxicity is a continuing concern, and analysis of this series has been non contributory in this respect. There is however, clear evidence from other studies which show that neurological sequelae are dose dependent (Ozols *et al.*, 1985, 1984; Grunberg *et al.*, 1989; Pollara *et al.*, 1988). Until the efficacy of possible neuroprotectors such as WR-2721 (Mollman *et al.*, 1988) have been confirmed, dose intensification with cisplatin will continue to be limited by neurotoxicity. Nevertheless our series shows that dose intensification can be employed without resultant debilitating neurotoxicity, and yet achieve similar responses to that reported in other series.

### References

BEHRENS, B.C., GROTZINGER, K.R., HAMILTON, T.C. & 8 others (1985). Cytotoxicity of 3 cisplatin analogues in a drug sensitive and a new cisplatin resistant human ovarian cancer cell line. *Proc. Am. Assoc. Cancer Res.*, **26**, 262.

GRUNBERG, S.M., SONKA, S., STEVENSON, L.L. & MUGGIA, F.M. (1989). Progressive parasthesia after cessation of therapy with very high-dose cisplatin. *Cancer Chemother. Pharmacol.*, **25**, 62–64.

HAINSWORTH, J.D., BURNETT, L.S., HOWARD, W.J.III, GROSH, W.W., JOHNSON, D.H. & GRECO, F.A. (1990). High-dose cisplatin combination therapy in the treatment of advanced epithelial ovarian carcinoma. *J. Clin. Oncol.*, **8**, 502–508.

KATZ, M.E., SCHWARTZ, P.E., KAPP, D.S. & LUIKART, S. (1981). Epithelial carcinoma of the ovary: current strategies. *Ann. Intern. Med.*, **95**, 98–111.

LAMBERT, H.E. & BERRY, R.J. (1985). High-dose cisplatin compared with high-dose cyclophosphamide in the management of advanced epithelial ovarian cancer (FIGO stages III and IV): report from the North Thames Cooperative Group. *Br. Med. J.*, **290**, 889.

MARKMAN, M., ROTHEMAN, R., HAKES, T. & 6 others (1991). Second-line platinum therapy in patients with ovarian cancer previously treated with cisplatin. *J. Clin. Oncol.*, **9**, 389–393.

MILLER, A.B., HOOGSTRATEN, B. & STAQUET, M. (1981). Reporting results of cancer treatment. *Cancer*, **47**, 207–214.

MOLLMAN, J.E., GLOVER, D., HOGAN, W.M. & FURMAN, R.E. (1988). Cisplatin Neuropathy: Risk factors, prognosis and protection by WR-2721. *Cancer*, **61**, 2192–2195.

OZOLS, R.F., OSTCHEGA, Y., MYERS, C.E. & YOUNG, R.C. (1985). High-dose cisplatin in hypertonic saline in refractory ovarian cancer. *J. Clin. Oncol.*, **3**, 1250–1256.

OZOLS, R.F., CORDEN, B.J., JACOB, J., WESLEY, M.N., OSTCHEGA, Y. & YOUNG, R.C. (1984). High-dose cisplatin in hypertonic saline. *Ann. Intern. Med.*, **100**, 19–24.

PANCINI, P.B., GREGGI, S., SCAMBIA, G., DIROBERTO, P., IACOBELLI, S. & MANCUSO, S. (1987). High-dose (200 mg $m^{-2}$) cisplatin induced neurotoxicity in primary advanced ovarian cancer patients. *Cancer Treat. Rep.*, **71**, 669–670.

POLLERA, C.F., MAROLLA, P., NARDI, M., AMEGLIO, F., COZZO, L. & BEVERE, F. (1988). Very high-dose cisplatin induced otoxicity: a preliminary report on early and long-term effects. *Cancer Chemother. Pharmacol.*, **21**, 61–64.

UICC, TNM. (1987). International Union Against Cancer, Geneva. *Classification of Malignant Tumours*, 3rd Edition.